# Association between secondhand smoke exposure and rheumatoid arthritis in US never-smoking adults: a cross-sectional study from NHANES

**DOI:** 10.1038/s41598-024-61950-2

**Published:** 2024-05-14

**Authors:** Xiaogang Qi, Junwen Fu, Jiaming Liu, Xupeng Wu, Xin Zheng, Shaowei Wang

**Affiliations:** 1 Department of Orthopedics, The Second Hospital of Shanxi Medical University, Taiyuan, 030000 China; 2 Department of Orthopedics, Yangquan Coal Group General Hospital, Yangquan, 045000 China; 3 Pain Department, Yangquan First People’s Hospital, Yangquan, 045000 China; 4 Department of Neurology, Changzhi Medical College Affiliated Heping Hospital, Changzhi, 046000 China; 5 Department of Cardiovascular Medicine, Yangquan First People’s Hospital, Yangquan, 045000 China

**Keywords:** Environmental sciences, Diseases, Rheumatology, Risk factors

## Abstract

While smoking is widely acknowledged as a risk factor for rheumatoid arthritis (RA), the connection between secondhand smoke (SHS) exposure and RA in never-smoking adults remains limited and inconsistent. This study aims to explore and quantify this association using serum cotinine levels. We conducted a cross-sectional study with 14,940 adults who self-report as never smokers, using National Health and Nutrition Examination Survey data from 1999 to 2018. Based on previous literature, SHS exposure was categorized into four groups according to serum cotinine levels. Compared to individuals in the unexposed group (serum cotinine < 0.05 ng/mL), the adjusted odds ratio (OR) for RA was 1.37 (95% CI 1.14–1.64, p = 0.001) in the low exposure group (serum cotinine at 0.05 to 0.99 ng/mL) after adjusting for covariates. However, no significant association was found in the moderate exposure group (serum cotinine at 1 to 10 ng/mL) or the heavy exposure group (serum cotinine ≥ 10 ng/mL). Furthermore, we detected a non-linear, positively saturated correlation between the cotinine levels after log2 transformation and RA, with a turning point at approximately − 2.756 ng/mL (OR = 1.163, 95% CI 1.073–1.261, p = 0.0002). The stability of the results was confirmed by subgroup analysis.

## Introduction

RA is characterized by its chronic, destructive, and incapacitating effects on joints^1^, with a worldwide prevalence of about 5 in 1000 adults^2^. It induces inflammation in the joints, potentially leading to irreversible joint impairment and functional disability, particularly in more severe cases^3^. While the exact cause of RA remains unclear, smoking has been identified as a significant factor in its onset^4–6^. In previous studies, the correlation between smoking and RA has been demonstrated^5,7^. Moreover, exposure to cigarette smoke has been associated with an elevated occurrence of RA, worsened disease progression, and reduced responsiveness to therapy in individuals^4,5^.

Indeed, exposure to SHS isn't any less risky than active smoking. SHS remains a significant public health concern. Reports indicate that approximately 1.3 million (with a range of 1.0–1.6 million) deaths worldwide were attributed to SHS in 2019 alone^8^. It is alarming that an estimated 37% of the global population is exposed to smoke from burning tobacco products or exhaled by smokers^9^. In the United States, statistics from 2015 to 2018 reveal that around 20.8% of nonsmoking adults aged 18 and older were exposed to SHS^10^. Although the adverse health effects of exposure to SHS are well-documented, including established links to elevated risks of various diseases such as cancer, cardiovascular diseases, respiratory diseases, type 2 diabetes, and even osteoporosis^9,11–14^, there is currently limited information available regarding its connection to the onset of RA. Acknowledged widely as a reliable and sensitive marker for recent tobacco smoke exposure, cotinine, a significant proximal metabolite of nicotine, precisely mirrors an individual's level of exposure within the preceding 72 h. It is now identified as a distinct chemical marker for assessing tobacco smoke exposure levels^15,16,17^.

Due to the limited and often incomplete clinical data available for assessing the association between SHS exposure and RA, we have chosen to utilize the NHANES database, which provides a substantial sample size and boasts high national representativeness, enhancing the robustness of our analysis. This cross-sectional study centered on non-smokers in the United States aims to explore the relationship between SHS exposure and the prevalence of RA, utilizing serum cotinine levels as a reliable indicator to quantify SHS exposure.

## Methods

### Study design and population

NHANES, overseen by the National Center for Health Statistics (NCHS), conducts a survey to assess the health and nutritional status of the noninstitutionalized population in the United States^18^. Employing a nationally representative sampling method, the survey utilizes stratified, multistage probability cluster sampling^19^. Approval for NHANES research protocols was obtained from the National Center for Health Statistics (NCHS) Research Ethics Review Board, and all participants provided written informed consent. No further Institutional Review Board approval was deemed necessary for this secondary analysis^20^. All methods of this study was performed in accordance with the principles outlined in the Declaration of Helsinki.

**Figure 1:**
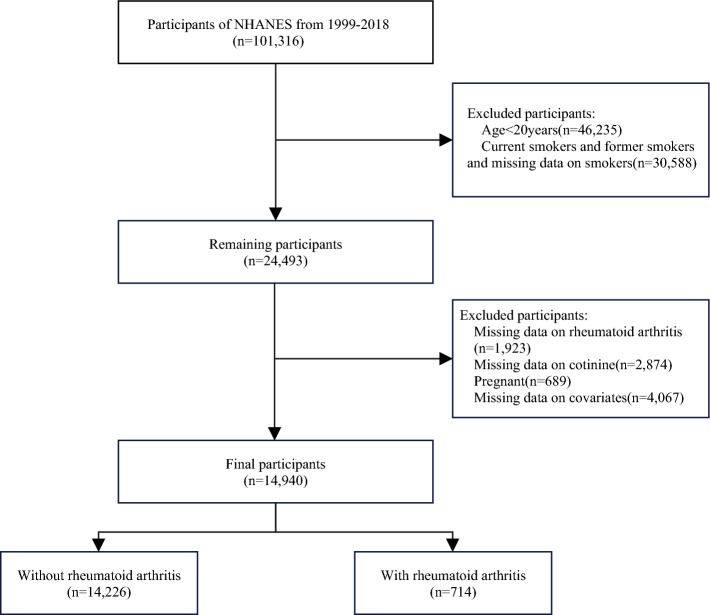
The study's flow diagram.

In this study, we analyzed NHANES data from 1999 to 2018, focusing on participants aged 20 or older who reported never smoking (n = 24,493). We excluded individuals who were pregnant (n = 689) and those with missing data on RA (n = 1,923), serum cotinine (n = 2,874), and other covariates (n = 4,067). Ultimately, our study sample comprised 14,940 participants. The participant selection process is visually depicted in Fig. 1.

### Smokers and RA assessment

In this study, never smokers were defined as participants who reported consuming fewer than 100 cigarettes in their lifetime and did not use any products containing nicotine in the past 5 days. Former smokers were participants who had a history of smoking over 100 cigarettes but were not presently engaged in smoking. Current smokers were participants who had smoked over 100 cigarettes in their lifetime and were actively smoking during the study. Participants were considered to have RA if they responded “RA” to the NHANES questionnaire questions, which asked whether a doctor had diagnosed them with arthritis and, if so, specified the type of arthritis.

### Tobacco exposure assessment

In accordance with findings from prior research^21–24^, and given that the majority of active smokers exhibit serum cotinine levels exceeding 10 ng/mL^15^, SHS exposure was classified into four categories: unexposed group (serum cotinine levels < 0.05 ng/mL), low SHS exposure group (0.05 ng/mL ≤ serum cotinine level < 1 ng/mL), and moderate SHS exposure group(1 ng/mL ≤ serum cotinine level ≤ 10 ng/mL), heavy SHS exposure group(serum cotinine level > 10 ng/mL). Serum cotinine measurement employed either isotope dilution-high performance liquid chromatography or atmospheric pressure chemical ionization tandem mass spectrometry^25^.

### Covariates

NHANES data collection involved administering a standardized participant questionnaire through household interviews, complemented by a comprehensive medical evaluation for each individual. In accordance with prior literature, the covariates taken into account in this study encompassed age, gender, marital status, race/ethnicity, educational level, family income, hypertension, diabetes, coronary heart disease, body mass index, physical activity, alcohol user, fish intake, asthma. Marital status was categorized as married, living with a partner, or living alone. Race/ethnicity included classifications such as non-Hispanic white, non-Hispanic black, Mexican American, or other races. Educational attainment was classified as less than 9 years, 9–12 years, and more than 12 years. Family income, based on the poverty income ratio (PIR), was grouped as low (PIR ≤ 1.3), medium (1.3 < PIR ≤ 3.5), and high (PIR > 3.5) according to a US government report^26^. Physical activity was classified into sedentary, moderate, and vigorous. Moderate activity involved at least 10 min of movement within the last 30 days, resulting in only light sweating or a mild to moderate increase in breathing or heart rate. Vigorous activity also required at least 10 min of movement within the last 30 days but resulted in profuse sweating or an increase in breathing or heart rate. Alcohol user was categorized as never, former, mild, moderate, or heavy drinker. The definitions of hypertension, diabetes, coronary heart disease, and asthma were based on questionnaire inquiries about whether participants had been informed by their doctor of these conditions in the past. The definition of fish intake is determined through dietary interview questionnaires, asking whether fish-containing foods have been consumed in the past 30 days. BMI was calculated through a standardized method of measuring height and weight.

### Statistical analysis

A secondary analysis was performed on publicly accessible datasets, categorical variables were represented as percentages (%), while continuous variables were expressed as means (SD) or median (IQR), according to the data distribution. To assess differences between groups, we employed analysis of variance, Kruskal–Wallis, and chi-squared tests. Additionally, to explore the relationship between SHS exposure and the occurrence of RA, we calculated odds ratios (OR) with 95 percent confidence intervals (95% CI) using statistical methods, such as multivariate logistic regression models. Model 1 underwent adjustments for sociodemographic characteristics, comprising age, gender, race/ethnicity, marital status, education level, and family income. Model 2 incorporated additional adjustments by including factors from Model 1, along with hypertension, diabetes, coronary heart disease, body mass index, physical activity, alcohol user, fish intake. Model 3 achieved full adjustment, encompassing factors from Model 2 and incorporating asthma.

After adjusting for covariates as specified in Model 3, a multivariate-adjusted restricted cubic spline analysis with four knots was employed to explore the potential nonlinear dose–response relationship between log2-transformed serum cotinine levels and RA.

In addition, after adjusting for covariates as specified in Model 3, a two-piecewise logistic regression model was used to explore the saturated relationship between log2-transformed serum cotinine levels and RA.

Possible alterations in this association were further explored based on age, gender, and BMI categories through multivariate logistic regression analysis. Likelihood ratio tests for interactions were incorporated. Consistent findings were observed even with the model adjustment through multivariate logistic and restricted cubic spline analyses.

All analyses were performed using the statistical software packages R (http://www.R-project.org, The R Foundation) and Free Statistics software version 1.9.2. Descriptive statistics were applied to characterize all participants, and two-tailed testing was utilized with a significance level of p < 0.05.

## Results

### Study population

From 1999 to 2018, a total of 14,940 individuals were successfully enrolled in this study (Fig. 1). Exclusions were applied to participants aged under 20 years (n = 46,235). We also excluded individuals who were pregnant (n = 689) and those with missing data on RA (n = 1,923), serum cotinine (n = 2,874), and other covariates (n = 4,067).

### Baseline characteristics

The baseline characteristics of participants, classified according to the presence or absence of RA, are depicted in Table 1. Among the 14,940 individuals, 714 (4.8%) had RA. The study participants had an average age of 46.7 ± 17.6 years, with 8,992 (60.2%) being female and 5,937 (39.7%) identifying as non-Hispanic White. In contrast to participants without RA, those with RA tended to be older, living alone, have a lower educational level, exhibit a lower family income, and experience a higher incidence of hypertension and diabetes.

**Table 1:** Baseline characteristics of 14,940 participants in the NHANES 1999–2018 cycles.

Characteristics	Total	Non-RA	RA
	(N = 14,940)	(N = 14,226)	(N = 714)
Age (years), Mean (SD)	46.7 ± 17.6	46.0 ± 17.4	60.8 ± 13.9
Gender, n (%)			
Male	5948 (39.8)	5745 (40.4)	203 (28.4)
Female	8992 (60.2)	8481 (59.6)	511 (71.6)
Marital status, n (%)			
Married or living with a partner	9255 (61.9)	8853 (62.2)	402 (56.3)
Living alone	5685 (38.1)	5373 (37.8)	312 (43.7)
Race/ethnicity, n (%)			
Non-hispanic white	5937 (39.7)	5677 (39.9)	260 (36.4)
Non-hispanic black	3022 (20.2)	2818 (19.8)	204 (28.6)
Mexican American	2781 (18.6)	2645 (18.6)	136 (19)
Others	3200 (21.4)	3086 (21.7)	114 (16)
Education level (years), n (%)			
<9	1405 ( 9.4)	1276 (9)	129 (18.1)
9–12	4528 (30.3)	4258 (29.9)	270 (37.8)
>12	9007 (60.3)	8692 (61.1)	315 (44.1)
Family income, n (%)			
Low	3930 (26.3)	3656 (25.7)	274 (38.4)
Medium	5591 (37.4)	5338 (37.5)	253 (35.4)
High	5419 (36.3)	5232 (36.8)	187 (26.2)
Physical activity, n (%)			
Sedentary	8261 (55.3)	7840 (55.1)	421 (59)
Moderate	3545 (23.7)	3368 (23.7)	177 (24.8)
Vigorous	3134 (21.0)	3018 (21.2)	116 (16.2)
Alcohol user, n (%)			
Never	3292 (22.0)	3080 (21.7)	212 (29.7)
Former	1985 (13.3)	1827 (12.8)	158 (22.1)
Mild	5343 (35.8)	5138 (36.1)	205 (28.7)
Moderate	2237 (15.0)	2163 (15.2)	74 (10.4)
Heavy	2083 (13.9)	2018 (14.2)	65 (9.1)
Hypertension, n (%)	3651 (24.4)	3279 (23)	372 (52.1)
Diabetes, n (%)	2332 (15.6)	2087 (14.7)	245 (34.3)
Coronary heart disease, n (%)	378 ( 2.5)	333 (2.3)	45 (6.3)
BMI (kg/m2), Mean (SD)	29.2 ± 7.0	29.1 ± 7.0	31.5 ± 7.3
Fish intake, n (%)	10,881 (72.8)	10,325 (72.6)	556 (77.9)
Asthma, n (%)	1893 (12.7)	1765 (12.4)	128 (17.9)
SHS exposure, n (%)			
Unexposed	10,449 (69.9)	9984 (70.2)	465 (65.1)
Low exposure	3725 (24.9)	3513 (24.7)	212 (29.7)
Moderate exposure	495 ( 3.3)	470 (3.3)	25 (3.5)
Heavy exposure	271 ( 1.8)	259 (1.8)	12 (1.7)

NHANES, National Health and Nutrition Examination Survey. RA, rheumatoid arthritis. BMI, body mass index. SHS, secondhand smoke.

### Association of SHS exposure with RA

Table 2 exhibits the results of multivariate logistic regression. Following adjustments for age, gender, marital status, race/ethnicity, educational level, and family income (model 1), an OR of 1.43 (95% CI 1.19–1.71, p < 0.001) was observed for the association between the low exposure group and RA. Following adjustments for the factors encompassed in model 1 and hypertension, diabetes, coronary heart disease, body mass index, physical activity, alcohol user, fish intake (model 2), consistency with model 1 was observed, with an OR of 1.36 (95% CI 1.13–1.63, p = 0.001). After adjusting for the factors included in model 2 and asthma, the association remained statistically significant, with an OR of 1.37 (95% CI:1.14–1.64) and the p for trend value was 0.001. Notably, a significant association with RA was observed exclusively in the low exposure group across all SHS exposure categories.

**Table 2:** Association between SHS exposure and RA among never-smoking adults in the NHANES 1999–2018 cycles.

	OR(95% CI)							
	Crude	p-value	Model 1	p-value	Model 2	p-value	Model 3	p-value
Log2-transformed cotinine, ng/mL	1.04(1.01–1.07)	0.008	1.06 (1.03–1.10)	< 0.001	1.06 (1.02–1.09)	< 0.001	1.06 (1.03–1.09)	< 0.001
SHS exposure								
Unexposed	1(Ref)		1(Ref)		1(Ref)		1(Ref)	
Low exposure	1.30 (1.10–1.53)	0.002	1.43 (1.19–1.71)	< 0.001	1.36 (1.13–1.63)	0.001	1.37 (1.14–1.64)	0.001
Moderate exposure	1.14 (0.76–1.73)	0.528	1.32 (0.85–2.05)	0.211	1.23 (0.79–1.92)	0.364	1.24 (0.79–1.94)	0.346
Heavy exposure	0.99 (0.55–1.79)	0.986	1.30 (0.70–2.40)	0.409	1.33 (0.72–2.46)	0.369	1.34 (0.72–2.48)	0.356
Trend test		0.043		0.001		0.006		0.005

SHS, secondhand smoke; RA, rheumatoid arthritis; NHANES, National Health and Nutrition Examination Survey; OR,odds radio;

CI, confidence interval; Ref, reference.

*Model1 adjusted for age, gender, marital status, race/ethnicity, educational level, family income.

Model 2 was adjusted for model 1 + hypertension, diabetes, coronary heart disease, body mass index, physical activity, alcohol user, fish intake.

Model 3 was adjusted for model 2 + asthma.

**Figure 2:**
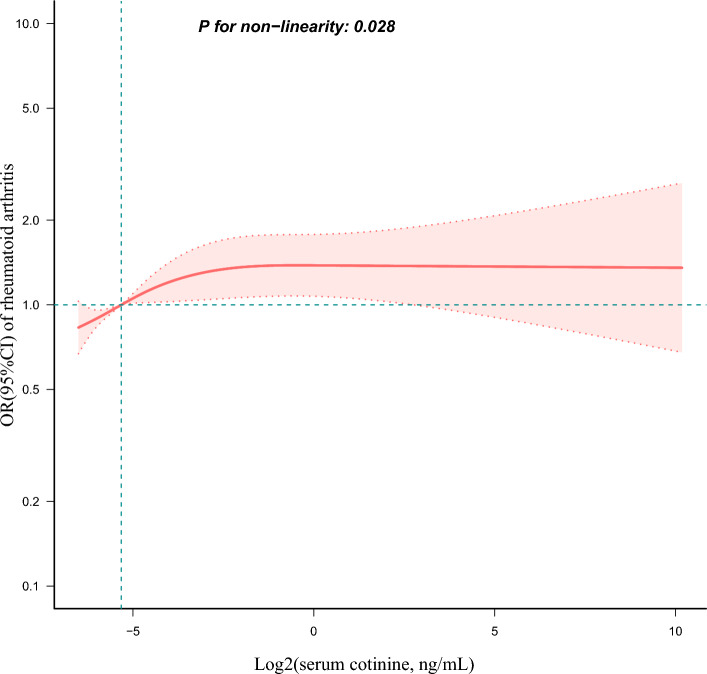
Associations between log2-transformed serum cotinine levels and the prevalence of rheumatoid arthritis. OR, odds ratio; CI, confidence interval. The restricted cubic spline model was adjusted for age, gender, marital status, race/ethnicity, educational level, family income, hypertension, diabetes, coronary heart disease, body mass index, physical activity, alcohol user, fish intake, asthma.

Moreover, a non-linear and positively saturated association was observed between log2-transformed serum cotinine levels and RA through restricted cubic spline analysis (p = 0.028 for non-linearity)(Fig. 2). In the threshold analysis, for individuals with log2-transformed serum cotinine levels below − 2.756 ng/mL, an OR of 1.163 (95% CI 1.073–1.261, p = 0.0002) was observed for each 1-unit increase in log2-transformed serum cotinine levels (ng/mL). However, no significant association was found between log2-transformed serum cotinine levels and RA in subjects with levels surpassing − 2.756 ng/mL, with an OR of 1.003 (95% CI 0.933–1.077) and a p-value of 0.943 (Table 3).

**Table 3:** Association between log2-transformed serum cotinine levels and RA using two-piecewise regression models.

Log2-transformed cotinine, ng/mL	Adjusted Model	
	OR (95%CI)	p-value
<− 2.756	1.163 (1.073–1.261)	0.0002
≥− 2.756	1.003 (0.933–1.077)	0.943
Log-likelihood ratio test		0.004

RA, rheumatoid arthritis; OR, odds ratio; CI, confidence interval.

*Adjusted for age, gender, marital status, race/ethnicity, educational level, family income, hypertension, diabetes, coronary heart disease, body mass index, physical activity, alcohol user, fish intake, asthma.

### Stratified analyses

**Figure 3:**
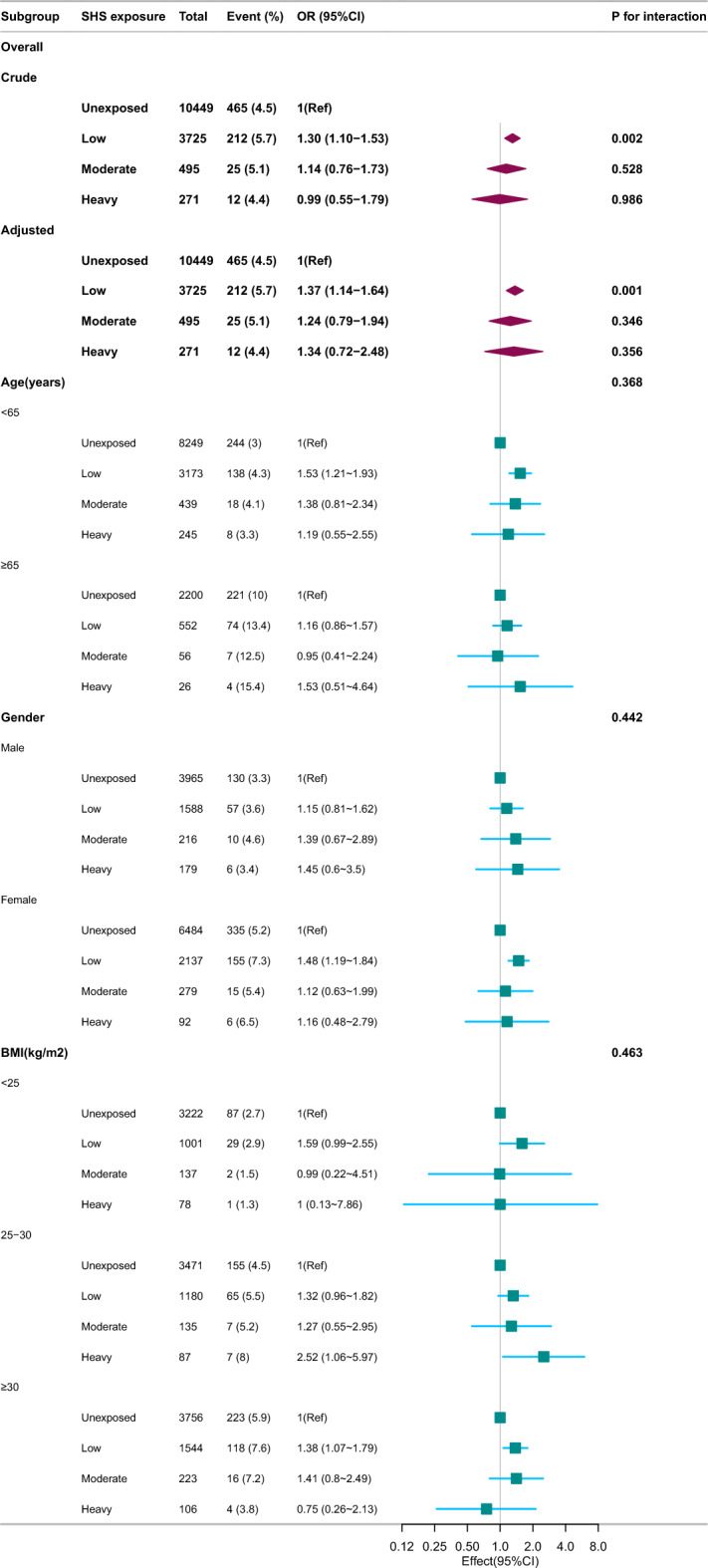
Stratified analysis of the relationship between SHS exposure and RA. Except for the stratification component itself, each stratification factor was adjusted for all other variables (age, gender, marital status, race/ethnicity, educational level, family income, hypertension, diabetes, coronary heart disease, body mass index, physical activity, alcohol user, fish intake, asthma). OR, odds ratio; CI, confidence interval.

No significant interactions were observed in any subgroup when stratified by age, gender, or BMI in the stratified analyses of multiple subgroups (Fig. 3). Similar results were obtained even after the model was adjusted through multivariate logistic and restricted cubic spline analyses.

## Discussion

In this large cross-sectional study, we focused on non-smokers in the United States and observed a non-linear, positively saturated relationship between SHS exposure and RA. When the log2-transformed serum cotinine levels were below − 2.756 ng/mL, each 1 ng/mL increase in log2-transformed serum cotinine levels corresponded to a 16.3% increase in the risk of developing RA. However, no significant correlation was found between SHS exposure and RA risk when the log2-transformed serum cotinine levels was exceeded − 2.756 ng/mL. Our study indicates that exposure to low levels of SHS increases the risk of developing RA, with a saturation effect observed between the two variables. Furthermore, the robustness of this relationship was also confirmed through subgroup analyses.

Previous investigations on the association between smoke exposure and RA are limited, and the results have been inconsistent. It was reported by Nguyen in a substantial prospective cohort study involving French women that an elevated risk of RA was associated with passive smoking exposure during childhood and/or adulthood, particularly in individuals who had never been active smokers^27^. Karen discovered a significant association between past and present tobacco use and the onset of RA in a prospective analysis involving 103,818 women, with a particular emphasis on seropositive RA^1^. While some investigations support a link between smoke exposure and RA, others contradict such a connection. No apparent relationship was observed between exposure to passive smoking and RA in a Swedish population-based case–control study^28^. Vanessa provides evidence contradicting the notion that passive smoking or an earlier age of smoking makes individuals more susceptible to RA^29^. However, no further study has been conducted to explore the relationship between SHS exposure and RA in never-smoking adults, utilizing serum cotinine to quantify the extent of SHS exposure. Serum cotinine levels can be employed to quantify exposure to smoking, enhancing the broad applicability of research findings. The NHANES presents a unique opportunity to investigate the potential association between SHS exposure and RA, as well as the dose–response relationship between the two. In contrast to previous studies, our research took a dual approach. We used self-reported criteria to identify non-smokers and employed serum cotinine levels to quantify SHS exposure. In our study, we observed that when log2-transformed serum cotinine levels surpassed a certain threshold, there was no significant association between SHS exposure and RA. However, below this threshold, an increase in serum cotinine levels correlated with a higher prevalence of RA. To validate our findings, further multicenter randomized controlled trials are imperative. Furthermore, research indicates that thirdhand smoke exposure, which refers to the inhalation of residual nicotine substances in the environment, can affect serum cotinine levels^30^. However, there is currently no universally accepted biomarker to effectively distinguish between thirdhand smoke exposure and SHS exposure. This indicates that there may be biases present when using serum cotinine levels to assess SHS exposure.

While the precise pathogenic impact of SHS exposure on RA remains unclear, various mechanisms have been suggested to enhance comprehension of the role of cigarette smoking in RA. The persistence of inflammation in RA may be linked to autoimmunity, characterized by oxidative stress, a proinflammatory condition, the production of autoantibodies, and epigenetic effects^7^. Researchers believed that tobacco exposure and its potential mechanisms in relation to RA involve various aspects as follows: Firstly, both the cellular and humoral components of the immune system are affected by cigarette smoking, resulting in a systemic proinflammatory condition^31,32^. Fibroblast-like synoviocytes affected by RA exhibit an induction of proinflammatory cytokines—such as IL-1a, IL-1B, IL-6, and IL-8, at both mRNA and protein levels—due to exposure to cigarette smoke condensate^33,34^. Secondly, cigarette smoke can trigger the release of endogenous free radicals^35^, compromising antioxidant systems and increasing oxidative stress, thereby contributing to the pathogenesis of RA^36^. Additionally, evidence presented by researchers from the Swedish Epidemiological Investigation of Rheumatoid Arthritis (EIRA) suggests that smoking could activate autoimmune responses specific to RA and citrullinated proteins^4^. Last but not least, smoking elevates the levels of Fas (CD95) and CD4 T-cells, making cells more prone to apoptosis. This heightened susceptibility to apoptosis results in increased levels of cellular debris, which might not be sufficiently eliminated in autoimmune disorders^37^. Smoking influences the immune system, resulting in diminished natural killer cells, suppressed hormonal cells, and cell-mediated immunity, along with the induction of dysfunction in T lymphocytes^38–42^. In a meta-analysis involving six cohorts, it was reported by De Rooy et al. that the impact of smoking on joint damage was mediated through ACPA^43^. In summary, tobacco exposure may contribute to or exacerbate rheumatoid arthritis through multiple pathways. Nevertheless, further prospective inquiries are required to authenticate the impact of SHS exposure on RA.

### Strengths and limitations

Our study’s strengths lie in the utilization of data from multiple survey cycles, which ensures a robust sample size. Coupled with its nationwide and multi-ethnic scope, our findings hold broad applicability and generalizability. Additionally, the dual approach-combining self-reported criteria for non-smoker identification and serum cotinine levels for quantifying SHS exposure-minimizes potential classification errors and subjectivity, significantly enhancing the study's overall reliability. However, our study does have limitations to consider. Firstly, being a cross-sectional study, it falls short of establishing causal relationships among dependent, independent, and covariate variables. Addressing this limitation requires the initiation of future prospective studies. Moreover, the relatively short half-life of serum cotinine restricts its ability to reflect long-term SHS exposure, instead emphasizing recent exposure. Additionally, exposure to thirdhand smoke can influence serum cotinine levels, potentially introducing bias when defining SHS exposure based on cotinine levels. Furthermore, the wide timeline of this study could potentially impact the obtained results, introducing the possibility of temporal bias. Despite the utilization of regression models and stratified analyses, it is crucial to acknowledge that complete elimination of residual confounding effects from unmeasured or unknown factors may not be attainable.

## Conclusion

In conclusion, our study centered on the never-smoking individuals in the United States and revealed a positively saturated relationship between SHS exposure and RA, indicating an inflection point of serum cotinine level after log2 conversion at approximately − 2.756 ng/mL. These findings aim to capture the attention of non-smokers. Moreover, further large-scale prospective investigations are necessary to confirm and extend our findings.

## Data Availability

The datasets generated or analysed during the current study are available from the corresponding author on reasonable request.
